# Supplementary material for: Learning to detect AI texts and learning the limits
Source: PLoS One. 2025 Oct 15;20(10):e0333007. doi: 10.1371/journal.pone.0333007 (PMC12527182; doi:10.1371/journal.pone.0333007)
Supplement: S2 File — All data and scripts used in the study, some additional descriptive statistics, scalable formate figures. (ZIP) [file pone.0333007.s002.tgz › data and scripts/OSF/scripts/analysis/Dependencies/GLS3_Assistant_vs_Is_correct.pdf]

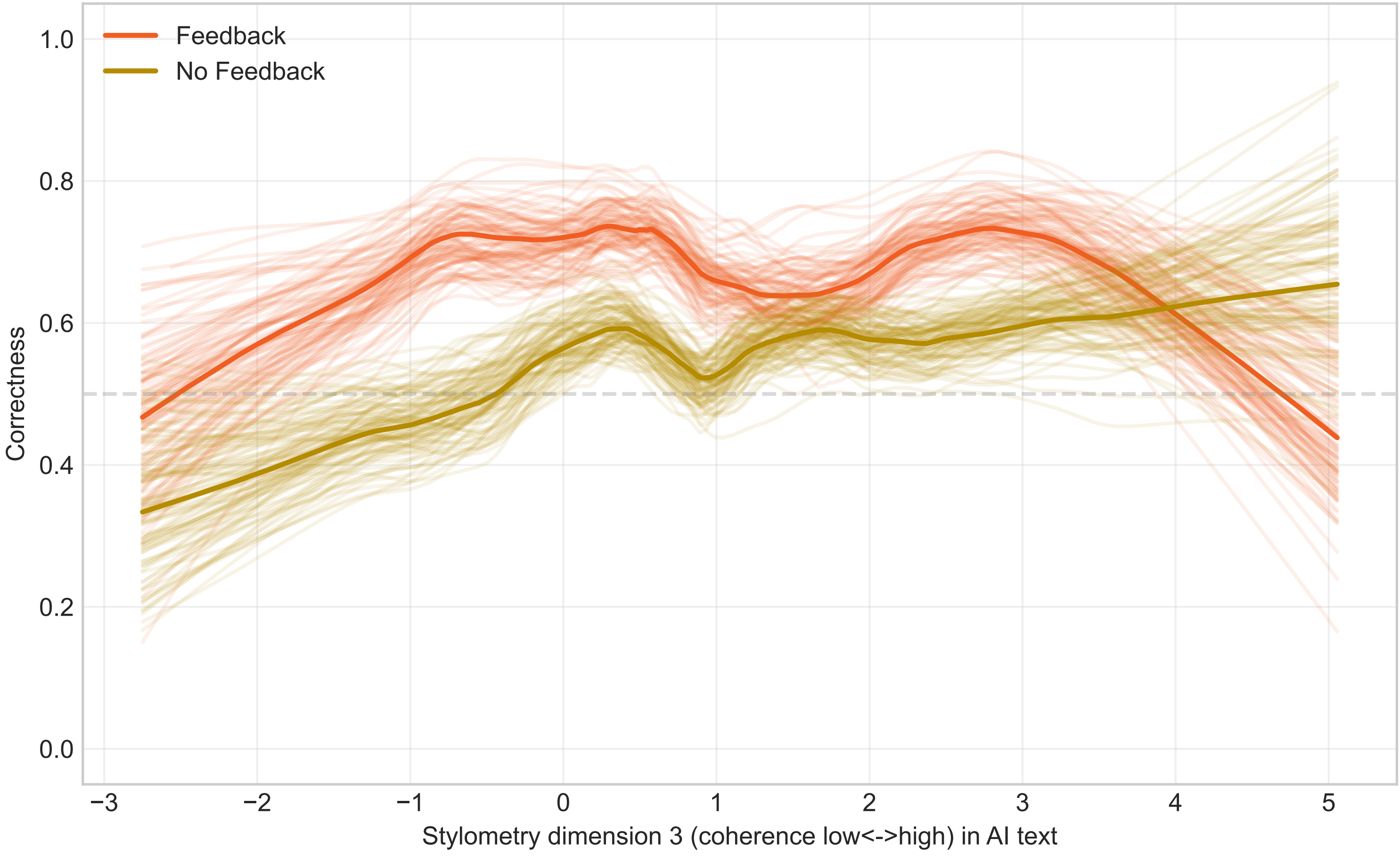

Feedback
No Feedback
Correctness
0.0
0.2
0.4
0.6
0.8
1.0
−3
−2
−1
0
1
2
3
4
5
Stylometry dimension 3 (coherence low<->high) in AI text
